# Electrochemical Oxidation of Primary Bile Acids: A Tool for Simulating Their Oxidative Metabolism?

**DOI:** 10.3390/ijms19092491

**Published:** 2018-08-23

**Authors:** Laura Navarro Suarez, Lea Brückner, Sascha Rohn

**Affiliations:** Hamburg School of Food Science, Institute of Food Chemistry, University of Hamburg, Martin-Luther-King-Platz 6, 20146 Hamburg, Germany; laura.gau@chemie.uni-hamburg.de (L.N.S.); lea.brueckner@studium.uni-hamburg.de (L.B.)

**Keywords:** bile acids, sterols, electrochemical oxidation, EC-MS

## Abstract

Bile acids are a subgroup of sterols and important products of cholesterol catabolism in mammalian organisms. Modifications (e.g., oxidation and 7-dehydroxylation) are predominantly exerted by the intestinal microbiota. Bile acids can be found in almost all living organisms, and their concentration and metabolism can be used for the assessment of the pathological and nutritional status of an organism. Electrochemical oxidation is a rapid, relatively inexpensive approach to simulate natural metabolic redox processes in vitro. This technique further allows the identification of oxidative degradation pathways of individual substances, as well as the demonstration of binding studies of generated oxidation products with biologically relevant molecules. When coupling an electrochemical and a high-resolution mass spectrometric system, oxidation products can be generated and identified directly by non-targeted ESI-MS. Here, a method for the generation of oxidation products of the primary bile acids cholic acid and chenodeoxycholic acid was exemplarily developed. Most products and the highest intensities were observed at a pH value of 6. For cholic acid, a high potential of 3 V was necessary, while for chenodeoxycholic acid, a potential of 2.4 V led to a higher number of oxidation products. In a second approach, a binding study with glutathione was performed to simulate phase II metabolism. It was possible to detect signals of free glutathione, free bile acids, and adducts of both reactants. As the resulting mass spectra also showed some new signals of the oxidized bile acid, which could not be observed without glutathione, it can be assumed that glutathione is able to bind reactive oxidation species before reacting with other products.

## 1. Introduction

Bile acids are essential metabolites that are involved in the emulsification and lipolysis of dietary fats and fat-soluble vitamins [1]. As signaling molecules, bile acids also detect and regulate not only their own concentration, but also that of cholesterol by activating various receptors and physiological pathways. In this way, they influence not only the regulation of lipids, but also of glucose and energy metabolism [1]. 

Bile acids are a group of sterol derivatives that are formed during the catabolism of cholesterol in liver hepatocytes. The following modifications lead the backbone of the bile acids: epimerization of the 3β-hydroxyl group, saturation of the double bond, and hydroxylation of the steroid backbone, as well as a side chain cutback and oxidation. Besides, there is a change in the configuration of the A-ring and the B-ring from trans to cis [2]. Thereby, all of the polar functional groups are on the same side, so that the molecule gets more amphiphilic. The resulting products are called primary bile acids [3]. After conjugation with glycine or taurine, conjugated bile acids are secreted into the gallbladder and enter the small intestine [4]. About 95% of the bile acids are absorbed along the terminal ileum and transported back to the liver via the portal vein, where they get reconjugated and reexcreted into the bile. This process is called enterohepatic circulation [5,6]. The amounts of bile acids that return to the liver via the enterohepatic circulation inhibit bile acid biosynthesis (from cholesterol) at a certain concentration by inhibiting the transcription of the enzyme CYP7A1 [1]. The part of the bile acids that is leaving the enterohepatic circulation is converted in the large intestine by the intestinal bacteria to secondary bile acids. This process begins in small parts in the small intestine, and is continued until it is almost complete in the colon [5]. In the conversion to secondary bile acids, a variety of reactions play an important role. Among the most important are the deconjugation, oxidation, and epimerization of the hydroxyl groups at C3, C7, and C12, 7-dehydroxylations, esterifications, and desulfations [6,7]. As a result, up to 20 different secondary bile acids can be detected in human feces. Those modifications of bile acid composition and the generation of secondary bile acids are performed by specific members of certain intestinal microbiota genera, e.g., Bacteriodes, Eubacterium, Lactobacillus, or Clostridium [8]. The endogenous mammalian synthesis and the composition of bile acids depend on many different parameters. These include the species, gender, individual genetic predisposition, and pathophysiological conditions. In addition, diet or medication also have an effect on bile acid biosynthesis [9]. As already mentioned, bile acids are subject to negative feedback regulation [9]. By binding to the farnesoid X receptor (FXR), a conformational change is accompanied so that it comes to a strong suppression of gene expression of the limiting enzyme CYP7A1 of the classical biosynthetic pathway through nuclear receptor cascade. In addition, an activated FXR is capable of stimulating the export pump. In this way, the homeostasis of toxic bile acids is ensured [10]. The literature described that a removal of the CYP7A1 gene leads to decreased bile acid synthesis, malnutrition, and the postnatal lethality of mice. This supports the assumption that the CYP7A1 gene plays an important role in the maintenance of bile acid and cholesterol homeostasis [11]. 

During recent years, interest in bile acid research has increased because of the correlation between their concentration and pathological and nutritional status of an organism [12,13]. Moreover, the influence and interactions between microbiome, diet, and bile acids are getting more and more into focus [13]. In the literature, there are some comprehensive studies dealing with this topic. Most of these studies were performed at the hand of animal experiments [14,15,16]. With the aim of replacing or supplementing these animal studies within the meaning of the European Commission Directive 2010/63/EU in the future, a common method might be the use of electrochemical oxidation. Still, metabolic studies have to be performed with laborious studies and analyses. Many studies deal with the imitation of drug metabolism [17,18]. For example, Baumann et al. investigated the application of electrochemistry coupled to mass spectrometry (EC-MS) for the determination of the metabolic pathway of tetrazepam in urine samples from patients, and demonstrated that the entire metabolic degradation of tetrazepam can be predicted by electrochemical simulation [19]. Another specific example is a study about the investigation of the biotransformation pathway of verapamil using electrochemistry coupled to mass spectrometry via a liquid chromatographic separation step (EC-LC-MS), where its biotransformation pathway was studied using a purely instrumental method based on EC-MS and compared to established in vitro approaches with liver microsomes [20]. Furthermore, there were also comprehensive studies dealing with the adduct formation of electrochemically generated intermediates and biomolecules. There, EC-MS was a useful tool for simulating oxidative drug metabolism and producing and characterizing the adduct formation of small molecules [21,22].

So far, an essential goal of most of those electrochemical studies is to get an idea to which extent the instrumental setup of EC-MS can simulate enzyme-catalyzed in vivo oxidation reactions [23]. Electrochemical oxidation offers a fast and purely instrumental approach that is less expensive and able to simulate one-electron processes. So, it seems to be possible to simulate selected redox processes of the cytochrome-P450 (CYP450) superfamily [24,25]. Another key advantage of EC-MS over traditional methods is the possibility of a direct study of reactive intermediates formed by oxidation reactions [26].

In electrochemistry, bile acids have not yet been frequently studied compounds, because of their inactivity under various conditions and electrode materials [27]. First attempts already showed the applicability of the oxidation of cholesterol to oxysterols using a boron-doped diamond electrode [28]. Another study dealt only with a determination of sterols, but did not consider the generation of oxidation products [29]. Steroid compounds and bile acids are electrochemically active only under specific conditions and the use of specific electrode material (e.g., mercury-based electrodes, or modified glassy carbon electrodes) [30]. A current study presented an approach of an anodic electrochemical oxidation of bile acids by activating the saturated steroid core by introducing double bonds via dehydration [31]. None of the studies so far determined the resulting oxidation products of the bile acids. Consequently, the aim of the present study was to simulate the metabolism of sterol derivatives by electrochemical methods, although they do not have an oxidizable double bond (such as cholesterol) in the sterane skeleton. The focus was to investigate to which extent a purely apparatus approach such as EC-MS is able to simulate the phase I and phase II metabolism of primary bile acids with a relatively simple experimental setup (compared to recent studies) using a Roxy™ potentiostat with a µPrepCell consisting of a boron-doped diamond electrode (Antec Leyden B.V., Leiden, The Netherlands). The chemical structure of formed oxidation products was characterized by a directly coupled mass spectrometric detection for a tentative structure elucidation. The online EC-MS method for the electrochemical generation and detection of oxidized bile acids should be optimized by means of different parameters such as solvents, pH value, and applied electric potential. For a simulation of phase II metabolism, the (follow-up) binding behavior of primary bile acids with endogenous biologically active conjugative molecules such as glutathione was studied as well.

## 2. Results

### 2.1. Electrochemical Oxidation of Primary Bile Acids by Means of EC-ESI-MS

**Figure 1:**
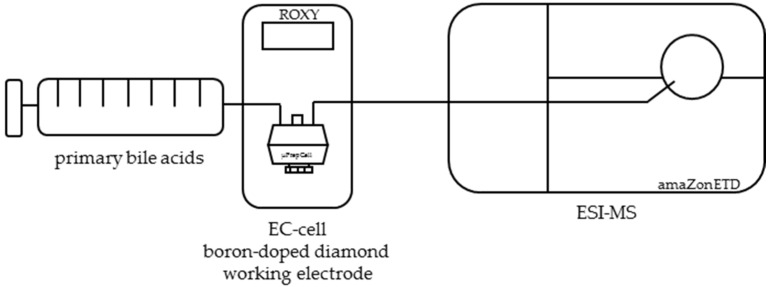
Instrumental setup for the electrochemical oxidation of primary bile acids using an EC-ESI-MS system. Primary bile acids are oxidized electrochemically via a thin layer cell consisting of a boron-doped working electrode (µPrepCell), and directly infused to the electrospray ionization mass spectrometer (ESI-MS).

In a first approach, the mobile phase and electrochemical parameters for the highest conversion rate of primary bile acids was assessed. For this purpose, the electrochemical oxidation of cholic acid (CA) and chenodeoxycholic acid (CDCA) was optimized by varying the pH value from pH 2 to pH 8 and the voltage from 0.3 to 3 V in steps of 0.3 V using a Roxy™ potentiostat with a µPrepCell consisting of a boron-doped diamond electrode (Antec Leyden B.V., Leiden, The Netherlands). Instrumental setup is shown in Figure 1.

**Figure 2:**
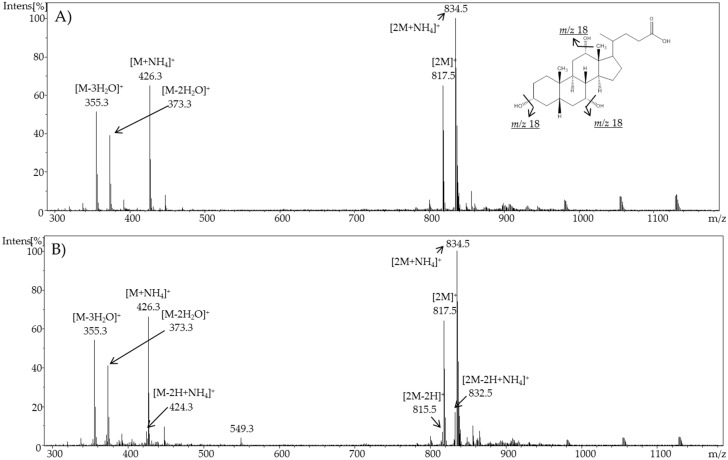
Full scan mass spectra of (**A**) pure cholic acid and (**B**) electrochemically oxidized cholic acid using a potential of ϕ = 3 V and at pH 6.

For cholic acid, a constant voltage increase (DC mode) from 2.1 V up to 3.0 V, and an acidic pH value led to an increasing formation of oxidation products. Consequently, 3 V was chosen as a potential for further experiments. In Figure 2, a comparison of the resulting mass spectra of cholic acid at 0 and 3 V and pH 6 is shown.

**Figure 3:**
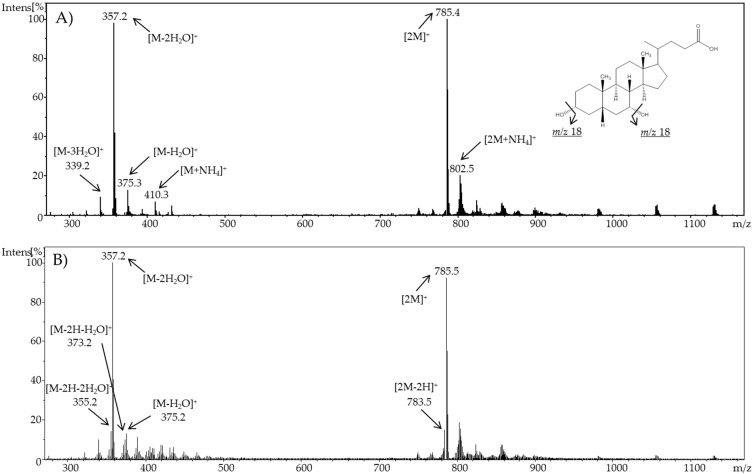
Full scan mass spectra of (**A**) pure chenodeoxycholic acid and (**B**) electrochemically oxidized chenodeoxycholic acid using a potential of ϕ = 2.4 V and at pH 6.

For chenodeoxycholic acid, a constant increase of voltage from 2.1 V up to 3.0 V (DC mode) and an acidic pH value led to an increasing formation of oxidation products. For that bile acid, 2.4 V was evaluated as being optimal for further experiments. In Figure 3, a comparison of the resulting mass spectra of chenodeoxycholic acid at 0 and 2.4 V is shown.

As expected, cholic acid and chenodeoxycholic acid formed quasi-molecular ions, as well as adducts with ammonia resulting from the mobile phase. In addition, neutral water loss is a universal reaction for bile acids in methanol in positive ESI ion mode [M + H-nH_2_O]^+^ [32]. The formation of dimers [2M]^+^ and trimers [3M]^+^ was also observed. The highest intensity in the resulting mass spectra of cholic acid was observed for the ammonia adduct of the dimer [2M + NH_4_]^+^ (*m*/*z* 834.5). When using a potential of 3 V, additional signals appeared. Mass differences of 2 Da suggested a successful oxidation. A decrease of the molecular mass by 2 Da indicates an oxidation of a hydroxyl group forming a keto group (e.g., for cholic acid: to 3-dehydrocholic acid (3-DHCA), 7-dehydrocholic acid (7-DHCA), or 12-dehydrocholic acid (12-DHCA), and for chenodeoxycholic acid: 3-ketolithocholic acid (3-KLCA), or 7-ketolithocholic acid (7-KLCA)). As these products are stereoisomers, the mass spectra alone are not meaningful for determining which compound is involved. Moreover, it can be also assumed that two hydroxyl groups in one molecule were oxidized, which can be supposed because of the decrease of 4 Da. 

### 2.2. Electrochemical Simulation of Endogenous Metabolism of Primary Bile Acids

**Figure 4:**
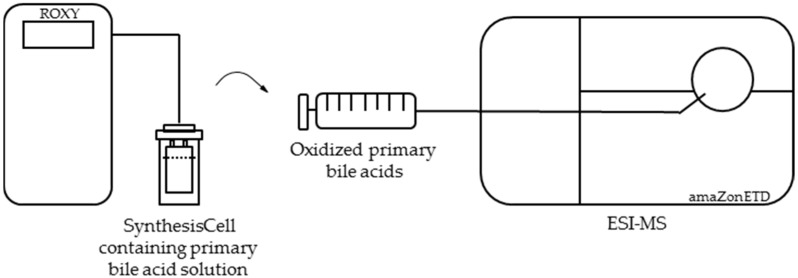
Instrumental setup for the simulation of the endogenous metabolism of primary bile acids. Bile acids are oxidized electrochemically via the synthesis cell. Manually collected aliquots were afterwards infused to the ESI-MS via a syringe pump.

To increase the yield for further investigations, a synthesis cell consisting of a boron-doped diamond electrode (SynthesisCell™, Antec Leyden B.V., Leiden, The Netherlands) was used. As the highest conversion rate was obtained at a slightly acidic pH value, a solution containing the primary bile acids cholic acid and chenodeoxycholic acid and pH 6 was used for this purpose. A constant potential of 3.0 V was used for the oxidation of cholic acid, while for chenodeoxycholic acid, a constant potential of 2.4 V was used. After every 30 min, an aliquot of the so far oxidized solution was taken from the reaction vessel and infused directly via a syringe pump into the mass spectrometer (cf. 4.3). For the instrumental setup, see Figure 4.

**Figure 5:**
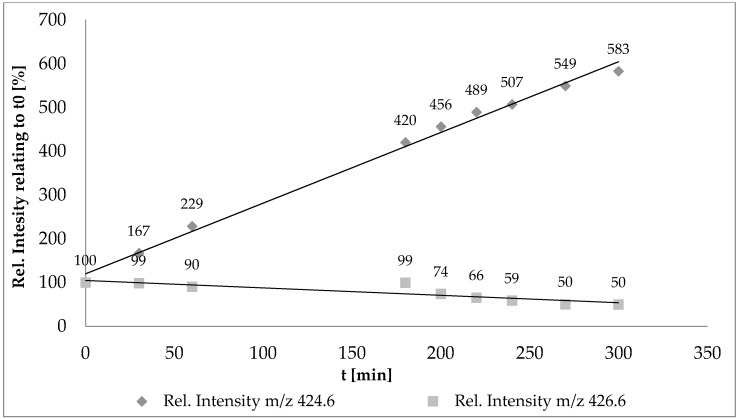
Reaction process of the electrochemical oxidation of primary bile acid cholic acid. *m*/*z* 424.6 represents oxidation products [M + NH_4_]^+^ while *m*/*z* 426.6 represents non-oxidized cholic acid [M-2H + NH_4_]^+^.

In Figure 5, only the results of the oxidation of cholic acid are illustrated because similar results were observed regarding chenodeoxycholic acid. It shows the relatively intensity of non-oxidized cholic acid (in relation to signalintesity at t0) representative by the ammonia adduct ([M + NH_4_]^+^ represented by m/z 426.6) and the relatively intensity of electrochemically oxidized cholic acid represented by the ammonia adduct ([M-2H + NH_4_]^+^ represented by m/z 424.6).

As shown in Figure 5, the relatively signal intensity of pure cholic acid decreased within the duration in the synthesis cell. After 300 min, only 50% of the original signal was left. Accordingly, the intensity of the signal of the oxidation products (*m*/*z* 424.6) increased. After 300 min, there was an ascent of 583% relative to the original signal.

**Figure 6:**
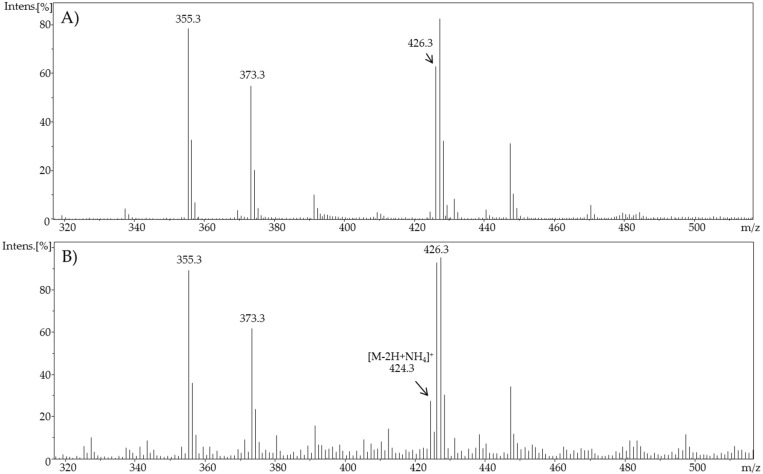
Full scan mass spectra of (**A**) non-oxidized cholic acid (ϕ = 0 V) and (**B**) electrochemically oxidized cholic acid (ϕ = 3 V) after 120 min in SynthesisCell™.

Figure 6 exemplarily shows a comparison of the full scan mass spectra of pure cholic acid (t0) and the full scan mass spectra of oxidized cholic acid after 120 min (t120). As already mentioned, an improvement toward a higher yield was successful. Similar results were observed during the oxidation of chenodeoxycholic acid. Consequently, the synthesis cell is able to generate higher quantities of electrochemically generated oxidation products for follow-up investigations.

To identify the potential generated oxidized products of the primary bile acid cholic acid, the solution was measured via LC-MS implementing a chromatographic separation of the target compounds, as it is not possible to make any statement about which compounds were synthesized exactly on the basis of the mass spectrometric data without separation. Using an advanced chromatographic separation of the oxidized sample prior to a mass spectrometric detection can give more reliable results about which oxidation products of the primary bile acids cholic acid, and chenodeoxycholic acid were generated. As known from the literature, the main oxidation products of cholic acids are 3-DHCA, 7-DHCA, and 7,12-diketolithocholic acid (7,12-DHCA), which are all commercially available for being used as reference compounds [6,7]. So, a standard solution containing these bile acids was first injected into the LC-ESI-MS system to compare the retention times of these references and the solution of oxidized cholic acid, and get a tentative chemical structure.

### 2.3. Investigation of Adduct Formation of Primary Bile Acids and Glutathione Using EC-ESI-MS

**Figure 7:**
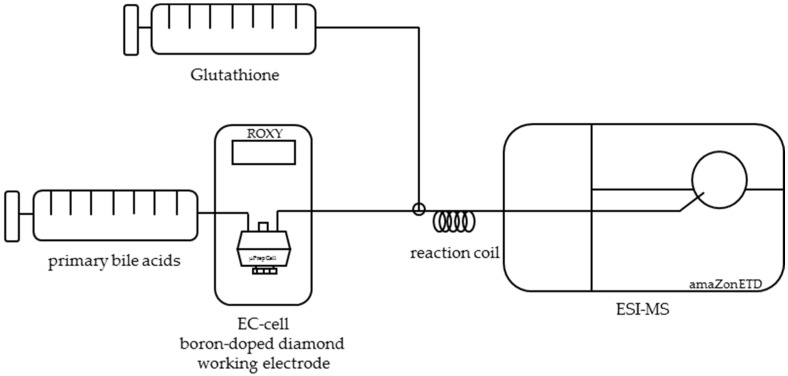
Instrumental setup for the simulation of phase II metabolism. Bile acids are oxidized electrochemically via a thin layer cell consisting of a boron-doped working electrode. A second flow system contained a solution of glutathione in methanol. Both solutions were merged in a three-way valve and left to react in a 100-µL coil. Afterwards, the mixture was infused into ESI-MS.

For evaluating the feasibility of simulating the phase II metabolism of primary bile acids and investigating the binding properties and the reactivity of the oxidation products, the reaction of the electrochemically oxidized primary bile acids cholic acid and chenodeoxycholic acid with glutathione was initiated via a reaction coil (Figure 7).

Glutathione (GSH) is an endogenous antioxidant and conjugation agent in the human organism [33]. It is present in high concentrations in all kinds of cells, and is therefore suitable as a representative biomolecule for performing a binding study. Glutathione can be chemically bound to bile acids by the reaction of a hydroxyl group of the bile acids with the thiol group of glutathione under the elimination of water, forming a thioether. Defined volumes of both reactants were injected into the electrochemical system via a syringe pump. Afterwards, the reaction products were directly detected using an ESI-MS ion trap mass analyzer in positive ion mode for identifying potential adduct formations. 

**Figure 8:**
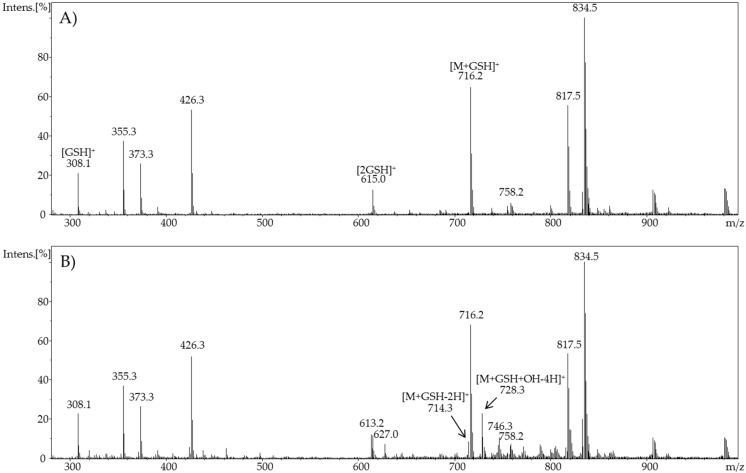
Full scan mass spectra of (**A**) the potential adduct formation of cholic acid and glutathione at 0 V (Solution pH 8) and (**B**) the potential adduct formation of cholic acid and glutathione in pulse mode (E1 = 3 V (t = 0,1 s), E2 = 0 V (t = 0,1 s)).

The resulting full scan mass spectra of the pure cholic acid and glutathione mixture, and the full scan mass spectra of oxidized cholic acid mixed with glutathione and an adjusted pulsed potential showed intense adduct formation (Figure 8), as well as new various results.

First, in the mass spectra of glutathione and pure cholic acid, signals of free glutathione, free cholic acid, and adducts of both reactants were observed. A high signal intensity was observed by the adduct of glutathione and cholic acid ([M + GSH]^+^, *m*/*z* 716.2). A second interesting observation was some new signals when supplying glutathione to oxidized cholic acid, which could not be observed without the supply of glutathione.

This suggests that glutathione is able to bind reactive oxidation species of cholic acid before reacting with other products. In particular of interest are the new signals with *m*/*z* of 728 and *m*/*z* 750. They are presumably adducts of glutathione and the twofold dehydrogenated cholic acid, which underwent aliphatic hydroxylation. It should be noted that no precise statement can be made about where the hydroxylation took place, as these compounds are stereoisomeric isobaric compounds. Additionally, mass spectra also show further signals whose origin and identity have not been clarified yet. 

## 3. Discussion

Due to the chemical structure and the fully saturated backbone, bile acids are known to be relatively inert for electrochemical oxidation compared to e.g., cholesterol [27]. In this study, a method with a simple setup that enables successfully electrochemical oxidation by using a constant (high) potential was introduced. Although the rate of yield of resulting oxidation products using an analytical cell was not as high as described in the literature [28], the products can be enriched when using a preparative synthesis cell. The limitation of this technique is given by relatively small yields and the resulting weak signals in the mass spectra. As a result, it remains difficult to gain enough substance for identifying the absolute structure of the oxidation products. As a consequence, the structures described herein are tentatively elucidated with a mass spectrometric approach or compounds that were commercially available as references.

The solvent used was a composition of methanol and double-distilled water with 20 mM of ammonium formate with different pH values. Weak acidic pH values promote the oxidation of bile acids. An optimal pH value for both cholic acid and chenodeoxycholic acid was pH 6. Similar results were also described in the literature for the electrochemical oxidation of cholesterol (as they used a solution of cholesterol in 90% MeOH containing 20 mmol/L ammonium formate) [28]. The use of perchloric acid as an oxidation reagent for promoting electrochemical oxidation did not increase the oxidation rate, as described in the literature [31]. However, it should be noted that the resulting oxidation products in recent studies were not measured via a mass spectrometer, but only through measuring the corresponding current. Also, the addition of a modifier (ammonium formate) did not improve the oxidation. Furthermore, it was recognized that an improvement of oxidation can be achieved by using optimized, pulsed potentials. The last-mentioned results should be contemplated in further experiments. Mass spectrometric detection was carried out directly in an online approach. It should be ensured that only the oxidation products generated in the electrochemical cell were detected. Any chemical changes of generated products due to storage or the loss of reactive products should be excluded.

**Figure 9:**
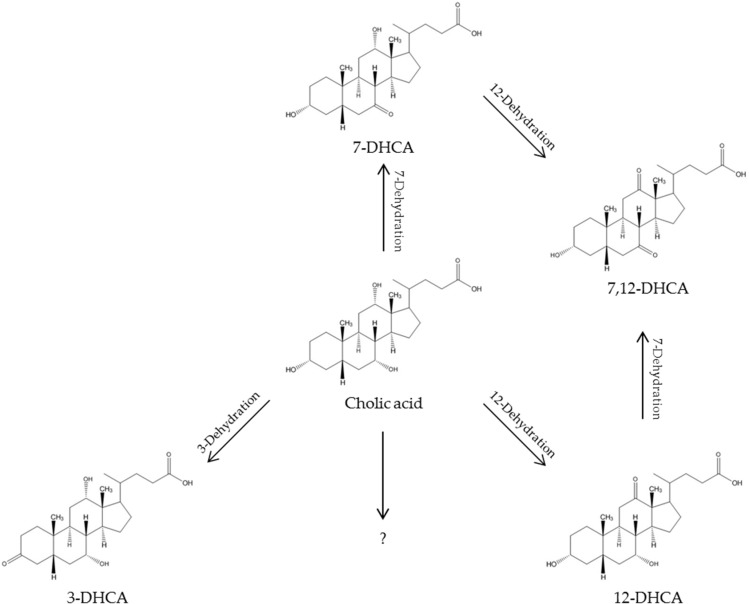
Observed phase I reactions of cholic acid.

The electrochemical oxidation of hydroxyl groups of primary bile acids leads to mono-, di-, or tricarbonyls. The oxidation of a hydroxyl group of cholic acid led to the oxidation products 3-dehydrocholic acid (3-DHCA), 7-dehydrocholic acid (7-DHCA), and/or 12-dehydrocholic acid (12-DHCA) (Figure 9). When using EC-MS without additional separation, these compounds cannot be distinguished, because they are stereoisomers or isobaric compounds. In the literature, it was reported that the reactivity of the several hydroxyl groups of the sterol skeleton decrease in the following order: C7 > C12 > C3 [34]. Therefore, a LC system was introduced into whole setup. The oxidation of two hydroxyl groups of cholic acid leads to 7,12-diketolithocholic acid (7,12-DHCA). An oxidation of all three hydroxyl groups in the molecule causes the formation of dehydrocholic acid (DHCA). However, not all metabolites were commercially available. Therefore, it was only possible to compare the oxidation products to a few reference compounds. In the present study, reactions leading to 7-DHCA, 12-DHCA, 3-DHCA, and 7,12-DHCA were observable, and the highest concentrations arose from 3-DHCA, which is different from the results found in the literature [34]. For a further structure elucidation, NMR is necessary. In the case of the cholic acid solution with a pH value of 8, high signal intensities of *m*/*z* 497 and *m*/*z* 549 could be observed in the mass spectrum. These *m*/*z* do not correspond to expected oxidation products. Presumably, these are fragmentation products of the sterol skeleton. Within pH 2, it was detectable that the primary bile acids underwent a reaction with methanol to a methyl ester (indicated by an increased *m*/*z* of 14 Da). It was able to slow down this reaction by cooling down the solutions. Nevertheless, it could be possible that storage in a solution of pH 2 lead to other products, which could disturb the mass spectrometric detection. The oxidation of chenodeoxycholic acid led to the oxidation products 3-ketolithocholic acid (3-KLCA), 7-ketolithocholic acid (7-KLCA), 7,12-diketolithocholic acid (7,12-DKLCA), and 3,12-diketolithocholic acid (3,12-DKLCA), which were compared to commercially available reference compounds and the tentative mass spectrometric structure elucidation (cf. 4.4).

As the electrochemical oxidation of a hydroxyl group to a keto group, i.e., simple dehydration, was carried out successfully, it can be recorded that it was possible to simulate the activity of hydroxysteroid dehydrogenases. Hydroxysteroid dehydrogenases (HSDH) are responsible for the cleavage of two protons in the molecule (simple hydrogenation). In the human organism, the epimerization of the hydroxyl groups occurs via stereospecific oxidation followed by the stereospecific reduction of the oxo groups [35]. In the human gut, microorganisms of the genera Bacteroides, Clostridium, Escherichia, Eggerthella, Eubacterium, Peptostreptococcus, and Rumniococcus are primarily responsible for these reactions [7]. MacDonald et al. postulated that the hydroxyl groups on dihydroxy-bile acids (e.g., deoxycholic acid (DCA), chenodeoxycholic acid (CDCA)) have been shown to be more prone to oxidoreductions than trihydroxy-bile acids such as cholic acid [36]. In this context, it would be interestingly to check whether that observation can be readjusted with the electrochemical approach presented herein. For the transformation of primary bile acid CA, it was necessary to adjust a higher potential (3.0 V) than for the oxidation of CDCA (2.4 V). To underline this hypothesis, an expansion toward other bile acids, e.g., DCA, would be important.

In blood plasma and liver metabolism, bile acid metabolites might be transformed further. Conjugation with glutathione is a possible way of conjugation. Glutathione can be chemically bound to bile acids by a reaction of a hydroxyl group of the bile acids with the thiol group of glutathione forming a thioester under the elimination of water [37]. So far, it was difficult to identify those adducts [38].

## 4. Materials and Methods

### 4.1. Chemicals

Cholic acid (CA) and ammonium formate were purchased from Sigma-Aldrich GmbH (Steinheim, Germany) and chenodeoxycholic acid (CDCA), dehydrocholic acid (DHCA), 7,12-diketolithocholic acid (7,12-DHCA), 7-dehydrocholic acid (7-DHCA), 12-dehydrocholic acid (12-DHCA), 3-dehydrocholic acid (3-DHCA), and 7-ketolithocholic acid (7-KLCA) were purchased from Steraloids Inc. (Newport, Rhode Island, USA). Methanol and acetonitrile were purchased from Carl Roth GmbH and Co. KG (Karlsruhe, Germany). Ammonium acetate was purchased from Merck KGaA (Darmstadt, Germany), and formic acid was purchased from VWR Chemicals (Darmstadt, Germany). All of the chemicals were used in the highest quality available. Water was purified before utilization via Direct-Q 3 UV-R system (Merck Millipore, Darmstadt, Germany).

### 4.2. Electrochemical Oxidation of Primary Bile Acids by Means of EC-ESI-MS

The electrochemical oxidation of primary bile acids was performed using a ROXY™ EC system (Antec Leyden B.V., Leiden, The Netherlands). This system was equipped with a preparative thin-layer cell (µPrepCell, Antec Leyden B.V., Leiden, The Netherlands) consisting of a boron-doped diamond working electrode, a titanium counter electrode, and a Pd/H_2_ reference electrode. Electrochemical potential was controlled using a Roxy™ potentiostat (Antec Leyden B.V., Leiden, The Netherlands). A solution containing 100 µM of the primary bile acids cholic acid, or chenodeoxycholic acid (in 90% methanol (*v*/*v*) and 10% purified and double-distilled water (*v*/*v*) with 20 mM of ammonium formate) was injected into the electrochemical system using an external syringe pump with a set flow rate of 50 µL/min. Four different solutions of varying pH values were prepared. The solutions were set to pH 2, pH 6, pH 7.4, and pH 8 to simulate the compartments of stomach, intestine, blood, and bile. The total volume of µPrepCell depending on an effective spacer thickness of 150 µm was 11 µL. The temperature of the electrochemical cell during analysis was 20 °C. First scan mode was applied (0 to 3 V in 0.3-V steps) to determine the electrochemical potential where the conversion rate was the highest. Afterwards, a constant potential was applied. The detection of oxidation products was performed with an ESI-MS ion trap mass analyzer in positive ion mode (amaZon speed ETD, Bruker Daltonics, Bremen, Germany), with the following mass spectrometer settings: ion spray voltage: 4.5 kV; ion source heater: 350 °C; source gas: 55 psi. For instrumental setup, see Figure 1. 

### 4.3. Electrochemical Simulation of Endogenous Metabolism of Primary Bile Acids

SynthesisCell™ (Antec Leyden B.V., Leiden, The Netherlands) was used to generate mg quantities of metabolites from primary bile acids, cholic acid, and chenodeoxycholic acid. The cell was equipped with a flat smooth magic diamond (BDD, 4.5 × 4.5 × 0.2 cm) working electode, a coiled platinum counter electrode, and a Pd/H_2_ reference electrode. Electrochemical potential was again controlled using a Roxy™ potentiostat (Antec Leyden B.V., Leiden, The Netherlands). 80 mL of a solution containing 2 mM of the primary bile acids cholic acid or chenodeoxycholic acid (in 90% methanol (v/v) and 10% purified and double-distilled water (v/v) with 20 mM of ammonium formate) was filled into the glass reaction vessel. The temperature of the electrochemical cell during oxidation was 20 °C. After placing the synthesis cell on the magnetic stirrer, a constant potential (which was identified in Section 4.2) was applied. Oxidation was monitored by the manual collection of 500-µL aliquot samples taken in 30-min intervals and manual injection via syringe pump into an ESI-MS ion trap mass analyzer in positive ion mode (amaZon speed ETD, Bruker Daltonics, Bremen, Germany), with the following mass spectrometer settings: ion spray voltage: 4.5 kV; ion source heater: 350 °C; source gas: 55 psi. For the instrumental setup, see Figure 4.

### 4.4. Identification of Oxidized Metabolites via LC-ESI-MS

For the determination of the type of oxidized bile acids that were generated in the synthesis cell, the solution containing the potentially oxidized species was measured via LC-MS. Chromatographic separation was performed using a Dionex UltiMate™ 3000 UHPLC system (Thermo Fisher Scientific Inc., Waltham, MA, USA) equipped with a Phenomenex^®^ reversed-phase HPLC column (Kinetex^®^ 2.6 µm RP 18 100 Å, 150 × 2.1 mm) and a Kinetex C18 security guard column (Phenomenex, Torrance, CA, USA) using a constant flow of 200 µL/min. Mass spectrometric detection was performed by an ESI-MS ion trap mass analyzer (amazon speed ETD, Bruker Daltonics, Bremen, Germany), recording mass spectra in a positive ion mode. The injection volume was 5 µL, the column oven temperature was set to 30 °C, and the autosampler was kept at 4 °C. The reversed phase chromatographic method consisted of a mobile phase system adapted from an existing method with some modifications for the optimized separation of oxidized bile acids [15,39]. The mobile phase A was water, and B was acetonitrile/methanol (3/1, v/v), both containing 0.1% formic acid and 10 mM of ammonium acetate. The gradient elution started with 70% A for 5 min, and then linearly decreased to 57.5% A within 5 min, which was kept constant for 2 min. Afterwards, composition was brought to 55% A in 3 min, which was held for 2 min. After decrease to 40% A linearly in 5 min, the percentage of A was first decreased further to 20% in 10 min, and directly after that to 0% in another 10 min. Composition was brought back to an initial ratio of 70% B within 2 min, followed by 10 min of re-equilibration. The LC-MS system was controlled by HyStar 3.2. Potential generated oxidation products were identified via retention time in comparison to commercial available bile acids. For this purpose, a multistandard solution containing bile acids DHCA, 7,12-DHCA, 7-DHCA, 12-DHCA, and CA was injected and analyzed via mass spectrometric detection. 

### 4.5. Investigation of Adduct Formation of Primary Bile Acids and Glutathione Using EC-ESI-MS

Phase II metabolism was simulated by the adduct formation of the primary bile acids cholic acid, chenodeoxycholic acid, and glutathione. For this purpose, a second flow system containing a glutathione solution with a concentration of 200 µM in methanol was supplied to the oxidized bile acid solution (see Section 4.2). The flow rate of both syringe pumps was set to 20 µL/min. The temperature of the electrochemical cell during oxidation was 20 °C. The ratio of both analytes in the reaction cell was 1:1. After a retention period in a reaction coil, the mixture was infused directly into an ESI-MS ion trap mass analyzer in positive ion mode (amaZon speed ETD, Bruker Daltonics, Bremen, Germany) with the following mass spectrometer settings: ion spray voltage: 4.5 kV; ion source heater: 350 °C; source gas: 55 psi. For the instrumental setup, see Figure 7.

## 5. Conclusions

Electrochemistry coupled to a mass spectrometric system was well used to investigate the phase I metabolism of the primary bile acids cholic acid and chenodeoxycholic acid. It was possible to develop conditions for the generation of oxidation products, although these metabolites, unlike cholesterol, do not have an easily oxidizable double bond, and are known to be relatively inert for electrochemical oxidation. The applicability of electrochemistry coupled with mass spectrometry for the investigation of the phase I metabolism of primary bile acids could be demonstrated. This method is able to supplement traditional methods as animal studies in the meaning of directive 2010/63/EU. Phase II metabolism was also successfully demonstrated, as a detection of the adducts of primary bile acids and glutathione was possible. A thorough clarification and quantification of the resulting chemical structures of the oxidation products and adducts was not possible at this time. However, the direct coupling to mass spectrometry allowed the suggestion of tentative structures. For elucidating absolute structures, a synthesis of higher amounts of reaction products in the synthesis cell for an analysis with nuclear magnetic resonance spectrometry (NMR) is recommended. In this context, it is also recommended to develop a method for separating and isolating the products. Using a preparative liquid chromatographic system should be suitable for this purpose. After that, absolute quantification using tandem mass spectrometry and the authentic standards will be possible for analyzing biological samples such as blood, bile, feces, and tissues as well. In this context, it can be mentioned that electrochemistry directly coupled to a mass spectrometry is not only able to simulate phase I and phase II metabolism, it is also a useful tool for the generation and tentative structure elucidation of further metabolites, because of being able to mimic CYP 450 catalyzed reactions [24].

## References

[1] Jenkins G., Hardie L.J. (2008). Bile Acids: Toxicology and Bioactivity.

[2] Ferdinandusse S., Denis S., Faust P.L., Wanders R.J. (2009). Bile acids: The role of peroxisomes. J. Lipid Res..

[3] Thomas C., Pellicciari R., Pruzanski M., Auwerx J., Schoonjans K. (2008). Targeting bile-acid signalling for metabolic diseases. Nat. Rev. Drug Discov..

[4] Vance D.E., Vance J.E. (2008). Biochemistry of Lipids, Lipoproteins and Membranes.

[5] Dawson P.A., Karpen S.J. (2015). Intestinal transport and metabolism of bile acids. J. Lipid Res..

[6] Ridlon J.M., Kang D.J., Hylemon P.B. (2006). Bile salt biotransformations by human intestinal bacteria. J. Lipid Res..

[7] Macdonald I.A., Bokkenheuser V.D., Winter J., McLernon A.M., Mosbach E.H. (1983). Degradation of steroids in the human gut. J. Lipid Res..

[8] Gerard P. (2013). Metabolism of cholesterol and bile acids by the gut microbiota. Pathogens.

[9] Chiang J.Y. (2004). Regulation of bile acid synthesis: Pathways, nuclear receptors, and mechanisms. J. Hepatol..

[10] Chiang J.Y. (2009). Bile acids: Regulation of synthesis. J. Lipid Res..

[11] Schwarz M., Lund E.G., Setchell K.D., Kayden H.J., Zerwekh J.E., Bjorkhem I., Herz J., Russell D.W. (1996). Disruption of cholesterol 7alpha-hydroxylase gene in mice. II. Bile acid deficiency is overcome by induction of oxysterol 7alpha-hydroxylase. J. Biol. Chem..

[12] Hofmann A.F. (1999). The continuing importance of bile acids in liver and intestinal disease. Arch. Int. Med..

[13] Worthmann A., John C., Ruhlemann M.C., Baguhl M., Heinsen F.A., Schaltenberg N., Heine M., Schlein C., Evangelakos I., Mineo C. (2017). Cold-induced conversion of cholesterol to bile acids in mice shapes the gut microbiome and promotes adaptive thermogenesis. Nat. Med..

[14] Faber H., Vogel M., Karst U. (2014). Electrochemistry/mass spectrometry as a tool in metabolism studies-A. review. Anal. Chim. Acta.

[15] Oberacher H., Pitterl F., Erb R., Plattner S. (2015). Mass spectrometric methods for monitoring redox processes in electrochemical cells. Mass Spectrom. Rev..

[16] Lohmann W., Baumann A., Karst U. (2010). Electrochemistry and LC-MS for Metabolite Generation and Identification: Tools, Technologies and Trends. Lc Gc Eur..

[17] Sayin S.I., Wahlstrom A., Felin J., Jantti S., Marschall H.U., Bamberg K., Angelin B., Hyotylainen T., Oresic M., Backhed F. (2013). Gut microbiota regulates bile acid metabolism by reducing the levels of tauro-beta-muricholic acid, a naturally occurring FXR antagonist. Cell Metab..

[18] Wegner K., Just S., Gau L., Mueller H., Gerard P., Lepage P., Clavel T., Rohn S. (2017). Rapid analysis of bile acids in different biological matrices using LC-ESI-MS/MS for the investigation of bile acid transformation by mammalian gut bacteria. Anal. Bioanal. Chem..

[19] Alnouti Y., Csanaky I.L., Klaassen C.D. (2008). Quantitative-profiling of bile acids and their conjugates in mouse liver, bile, plasma, and urine using LC-MS/MS. J. Chromatogr..

[20] Jurva U., Wikstrom H.V., Bruins A.P. (2000). In vitro mimicry of metabolic oxidation reactions by electrochemistry/mass spectrometry. Rapid Commun. Mass Spectrom..

[21] Frensemeier L.M., Buter L., Vogel M., Karst U. (2017). Investigation of the oxidative transformation of roxarsone by electrochemistry coupled to hydrophilic interaction liquid chromatography/mass spectrometry. J. Anal. Atom. Spectrom..

[22] Baumann A., Lohmann W., Schubert B., Oberacher H., Karst U. (2009). Metabolic studies of tetrazepam based on electrochemical simulation in comparison to in vivo and in vitro methods. J. Chromatogr. A.

[23] Jahn S., Baumann A., Roscher J., Hense K., Zazzeroni R., Karst U. (2011). Investigation of the biotransformation pathway of verapamil using electrochemistry/liquid chromatography/mass spectrometry—A comparative study with liver cell microsomes. J. Chromatogr. A.

[24] Buter L., Vogel M., Karst U. (2015). Adduct formation of electrochemically generated reactive intermediates wi th biomolecules. Trac-Trend Anal. Chem..

[25] Plattner S., Erb R., Pitterl F., Brouwer H.J., Oberacher H. (2012). Formation and characterization of covalent guanosine adducts with electrochemistry-liquid chromatography-mass spectrometry. J. Chromatogr. B Anal. Technol. Biomed. Life Sci..

[26] Pecková K., Nesmerák K. (2012). Electrochemistry of Bile Acids, Cholesterol, and Related Compounds (An Overview). Sens. Electroanal..

[27] Weber D., Ni Z.X., Vetter D., Hoffmann R., Fedorova M. (2016). Electrochemical oxidation of cholesterol: An easy way to generate numerous oxysterols in short reaction times. Eur. J. Lipid Sci. Technol..

[28] Bussy U., Boisseau R., Thobie-Gautier C., Boujtita M. (2015). Electrochemistry-mass spectrometry to study reactive drug metabolites and CYP450 simulations. Trac-Trend Anal. Chem..

[29] Kotani A., Hakamata H., Nakayama N., Kusu F. (2011). Picomole Level Determination of Cholesterol by HPLC with Electrochemical Detection Using Boron-doped Diamond Electrode after Performance Assessment Based on the FUMI Theory. Electroanal.

[30] Klouda J., Barek J., Nesmerak K., Schwarzova-Peckova K. (2017). Non-Enzymatic Electrochemistry in Characterization and Analysis of Steroid Compounds. Crit. Rev. Anal. Chem..

[31] Klouda J., Barek J., Kočovský P., Herl T., Matysik F.-M., Nesměrák K., Schwarzová-Pecková K. (2018). Bile acids: Electrochemical oxidation on bare electrodes after acid-induced dehydration. Electrochem. Commun..

[32] Qiao X., Ye M., Liu C.F., Yang W.Z., Miao W.J., Dong J., Guo D.A. (2012). A tandem mass spectrometric study of bile acids: Interpretation of fragmentation pathways and differentiation of steroid isomers. Steroids.

[33] Njalsson R., Norgren S. (2005). Physiological and pathological aspects of GSH metabolism. Acta Paediatr..

[34] Nair P.P. (1971). The Bile Acids Chemistry, Physiology, and Metabolism.

[35] Hylemon P.B., Harris S.C., Ridlon J.M. (2018). Metabolism of hydrogen gases and bile acids in the gut microbiome. FEBS Lett..

[36] Macdonald I.A., Williams C.N., Mahony D.E. (1974). A 3 alpha- and 7 alpha-hydroxysteroid dehydrogenase assay for conjugated dihydroxy-bile acid mixtures. Anal. Biochem..

[37] Mitamura K., Sogabe M., Sakanashi H., Watanabe S., Sakai T., Yamaguchi Y., Wakamiya T., Ikegawa S. (2007). Analysis of bile acid glutathione thioesters by liquid chromatography/electrospray ionization-tandem mass spectrometry. J. Chromatogr. B.

[38] Hofmann A.F., Hagey L.R. (2008). Bile acids: Chemistry, pathochemistry, biology, pathobiology, and therapeutics. Cell. Mol. Life Sci..

[39] Cai X.H., Liu Y.D., Zhou X., Navaneethan U., Shen B., Guo B.C. (2012). An LC-ESI-MS method for the quantitative analysis of bile acids composition in fecal materials. Biomed. Chromatogr..

